# Creating Evidence-Based Youth Mental Health Policy in Sub-Saharan Africa: A Description of the Integrated Approach to Addressing the Issue of Youth Depression in Malawi and Tanzania

**DOI:** 10.3389/fpsyt.2019.00542

**Published:** 2019-08-28

**Authors:** Stanley Kutcher, Kevin Perkins, Heather Gilberds, Michael Udedi, Omary Ubuguyu, Tasiana Njau, Rex Chapota, Mina Hashish

**Affiliations:** ^1^Department of Psychiatry, IWK Health Centre and Dalhousie University, Halifax, NS, Canada; ^2^Farm Radio International, Ottawa, ON, Canada; ^3^Malawi Ministry of Health, Lilongwe, Malawi; ^4^Muhimbili National Hospital, Dar es Salaam, Tanzania; ^5^Muhimbili University of Health and Allied Sciences, Dar es Salaam, Tanzania; ^6^Farm Radio International, Lilongwe, Malawi

**Keywords:** adolescent depression, radio, social and behavior change communication, school-based mental health, primary care, low-income country, sub-Saharan Africa

## Abstract

Addressing depression in young people is a health-care policy need in sub-Saharan Africa. There exists poor mental health literacy, high levels of stigma, and weak capacity at the community level to address this health-care need. These challenges are significant barriers to accessing mental health care for depression, soon to be the largest single contributor to the global burden of disease. We here describe an innovative approach that addresses these issues simultaneously while concurrently strengthening key mental health components in existing education and health-care systems as successfully applied in Malawi and replicated in Tanzania. Improving the pathway to care for young people with depression requires the following: improving mental health literacy (MHL) of communities, youth, and teachers; enhancing case identification and linking schools to community health clinics; improving the capacity of community health-care providers to identify, diagnose, and effectively treat depression in youth. Funded by Grand Challenges Canada, we developed and applied a program called “An Integrated Approach to Addressing the Challenge of Depression Among the Youth in Malawi and Tanzania” (IACD). This was an example of, a horizontally integrated pathway to care model designed to be applied in low-resource settings. The model is designed to 1) improve awareness/knowledge of mental health and mental disorders (especially depression) in communities; 2) enhance mental health literacy among youth and teachers within schools; 3) enhance capacity for teachers to identify students with possible depression; 4) create linkages between schools and community health clinics for improved access to mental health care for youth identified with possible depression; and 5) enhance the capacity of community-based health-care providers to identify, diagnose, and effectively treat youth with depression. With the use of interactive, youth-informed weekly radio programs, mental health curriculum training for teachers and peer educators in secondary schools, and a clinical competency training program for community-based health workers, the innovation created a “hub and spoke” model for improving mental health care for young people. Positive results obtained in Malawi and replicated in Tanzania suggest that this approach may provide an effective and potentially sustainable framework for enhancing youth mental health care, thus providing a policy ready framework that can be considered for application in sub-Saharan Africa.

## Introduction

Mental disorders account for the highest burden of disease among young people worldwide, with depression soon poised to become the largest single contributor to the burden of disease globally (1, 2). About 70% of mental disorders can be diagnosed before the age of 25, making the adolescent years a critical time for mental health promotion, early identification, and rapid access to effective mental health care (3–7). If left untreated, depression can contribute to early mortality and increased morbidity and has a significant negative impact on quality of life and future vocational success for young people (8). The economic dividend for early identification and effective treatment of depression in Low and Middle Income Countries (LMICs) is considerable (9).

However, numerous challenges exist that limit rapid access to effective care for young people with depression. While there is a paucity of epidemiological data on the prevalence of depression in young people in sub-Saharan Africa (10, 11), the World Health Organization estimates that approximately 6–8% of young people live with depression (1, 12). While research about youth mental health is scant in Malawi and Tanzania, available studies indicate that depression is a common disorder. Udedi (13) found a prevalence rate of roughly 30% in attendees of the Matawade Health Center in Zomba, and Kauye et al. (14) reported a rate of 19% in attendees of other clinics. In a study of pregnant women and young mothers (many of whom are teenagers), Stewart et al. (15) reported rates of depression ranging between 10.7% and 21.1%. Kim et al. (16) report a depression rate of 20% in adolescents attending HIV/AIDS clinics. In Tanzania, the 2008 Global School-Based Student Health Survey (GSHS) reported that 23.6% of students felt sad, lonely, or hopeless daily, with 11.2% reporting suicidal thoughts (17). Similar rates have been reported in Nigeria (18) and Kenya (19).

Furthermore, Crabb et al. (20) report that there is poor understanding of mental health and mental illness throughout sub-Saharan Africa (SSA), and in many countries in SSA, mental disorders such as depression are often not recognized as an illness and remain largely untreated (20, 21). Numbers of highly trained specialty providers are very low, and availability of effective mental health care in community clinics is limited (6, 14, 22, 23).

### Settings

Malawi is a low-income country of about 16 million people in Eastern SSA with annual health spending per capita of about $43.00 and about 2% of the overall health budget spent on mental health services (24, 25). Mental health legislation was revised in 2005. There is no mental health policy, although one is under development. Some community health providers have received limited training in mental health care in the past 5 years and none in youth mental health. The ratio of mental health professionals to population is about 2.5/100,000, with the majority being psychiatric nurses (20).

The United Republic of Tanzania is a low-income country, with about 45 million people in eastern SSA (24–26) currently listed as one of the 49 least-developed countries in the world (Development Policy and Analysis Division, DESA). The total government expenditure on health per capita is $42.00 (27), with mental health expenditures comprising 2.4% of the total health budget (ibid). A mental health policy and plan (2006) is available and currently undergoing revision. Very few primary health-care providers have received training in mental health care and none in youth mental health. Psychiatric nurses provide most of the mental health services with a ratio of 2/100,000 but with limited training in youth mental health. Mental health coordinators with no training in youth mental health are assigned to districts, with 94% of the 121 districts having mental health coordinators (28).

**Figure 1:**
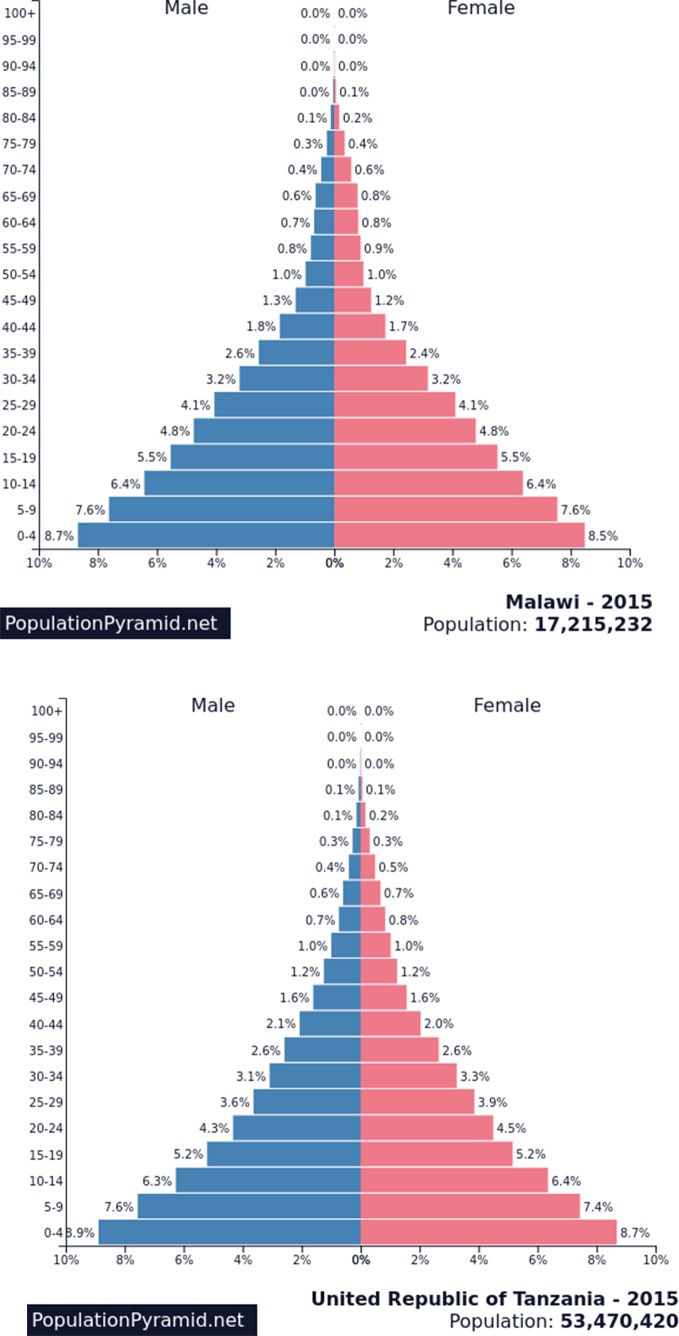
Current population age pyramids: Malawi and Tanzania.

In contrast to the severely limited capacity to meet youth mental health-care needs in Malawi and Tanzania, over 50% of the current population is below 20 years of age (see **Figure 1**). Due to improvements in age of mortality, as this cohort ages, it will bring with it increasing demands for mental health care to a system that is already struggling to meet current needs. Thus, there is urgent need to develop effective and frugal mental health policies, plans, and the capacity to address these needs, as effective interventions in this age group can be expected to show positive results in the present and into the future.

The above considerations identify the need for the development, application, and evaluation of an effective and frugal mental health policy and programmatic framework that can simultaneously improve mental health literacy and enhance capacity for improved access to effective mental health care for young people with depression. The “An Integrated Approach to Addressing the Challenge of Depression Among the Youth in Malawi and Tanzania (IACD)” was designed to meet that need. The IACD innovation promotes the development of an integrated horizontal pathway to mental health care for youth, beginning with enhancing MHL in both the community (through interactive radio programs and other electronic interventions, such as Facebook and WhatsApp) and schools (through teacher training, curriculum MHL resources, and school-based radio-listening clubs). The innovation builds capacity for early identification of depression by teachers and community health-care providers and links schools to community health clinics to help enhance access to mental health care. Community health-care providers are trained in the identification, diagnosis, and evidence-based treatment for depression in young people. Applied together, these linked components create a pathway to care for youth with depression.

This innovative combination of interactive radio programs, school-based MHL, and community clinic interventions was the result of a cross-disciplinary collaboration between Canadian communications (Farm Radio International) and academic (Dr. Stan Kutcher, Professor of Psychiatry at Dalhousie University, Halifax Canada) expertise. In Malawi, Farm Radio Trust implemented the radio-based activities, and the World University Service of Canada (WUSC) and the Malawi Ministry of Health led the application of training interventions and data collection. In Tanzania, Farm Radio International’s Tanzania country office implemented the radio-based activities and provided logistical support for training and research activities supported by the Ministry of Health and Social Welfare.

**Figure 2:**
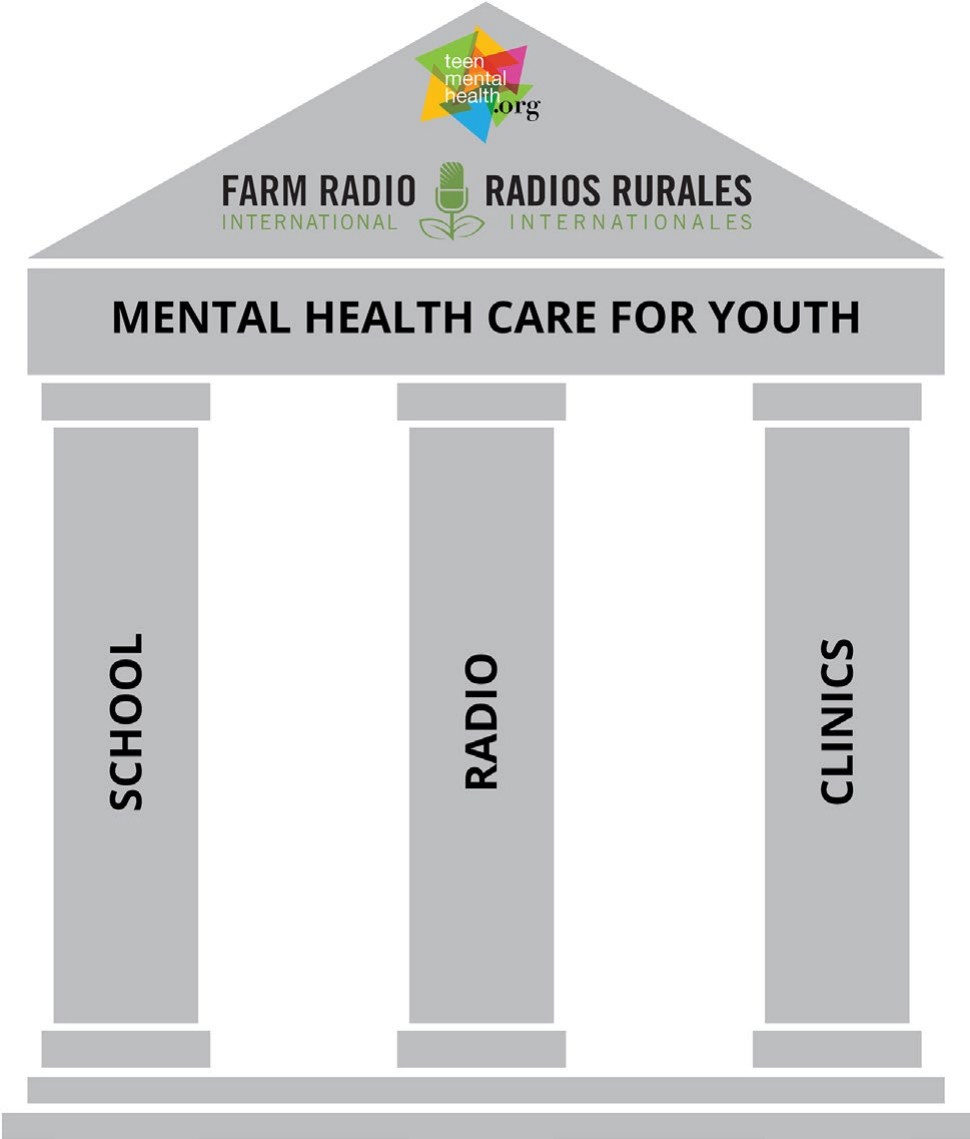
The horizontally integrated pathway for youth mental health care for sub-Saharan Africa.

Visually, this is illustrated in the three-legged stool diagram (see **Figure 2**) where the seat of the stool is the horizontally integrated pathway to youth mental health care and the supporting legs are 1) youth-participant interactive radio programs that address mental health awareness; 2) school-based mental health literacy and capacity building for early identification in schools; and 3) training for community health workers to enhance their ability to diagnose and effectively treat depression in young people.

As this innovation was applied, a variety of research approaches to determine the impact of the various components were used. These included mixed-methods quantitative and qualitative data collection, prospective longitudinal cohort, and cluster controlled designs (see **Table 1**). The interventions were first applied in Malawi and then replicated in Tanzania. This tiered approach addresses the issue of potential scalability of the innovation, as positive results obtained in Malawi and replicated in Tanzania indicate that it is less likely to be nation specific and more likely to be generalizable to multiple settings. The interventions were applied in Central Malawi (Lilongwe, Salima, and Mchinji districts) and in Northern Tanzania (Meru District Centre and Arusha District Centre). All research activities received appropriate ethics approval in their respective jurisdictions.

**Table 1:** Program designs for radio program, school setting and community healthcare settings implementations.

Radio program	School settings	Community health-care settings
Semi-controlled cluster design cross-sectional comparison—mental health awareness and mental health literacy	Prospective cohort design—teachers’ mental health literacy	Prospective cohort design—provider competencies (knowledge; attitudes; confidence)
Prospective controlled cluster design—mental health awareness and mental health literacy	Prospective cohort design—peer educator mental health literacy	Clinical outcomes—screening rates; diagnosis methods and rates; treatments used; patient outcomes using standardized measures
	Cross-sectional XXXX design—teacher reports on impact of intervention on student outcomes	
	Cross-sectional responder interviews—numbers of students approaching teachers for mental health concerns; numbers of students referred by schools to community health-care centers	

With the exception of the peer mental health educator component, which was undertaken under the leadership of Dr. Hamawaka (deceased) and the Guidance, Counselling and Youth Development Centre for Africa and not conducted in Tanzania, the Tanzania sites were a replication of the Malawi intervention.

### Mental Health Literacy as a Foundation for Enhancing Mental Health Outcomes (Radio Drama for Community- and Curriculum-Based Approaches for Schools)

Mental health literacy is an important component of health literacy and is essential for improving access to mental health care and reducing stigma related to mental illness (29–35). MHL is foundational for improving mental health outcomes and includes four related components: 1) enhancing capacity to obtain and maintain good mental health; 2) enhancing understanding of mental disorders and their treatments; 3) decreasing stigma related to mental illness; and 4) enhancing help-seeking efficacy (33–35).

Improving MHL can be addressed at both community and institutional levels. In communities, interventions that raise mental health awareness and improve knowledge can be applied. Youth popular mass media (such as radio programs) may provide a useful vehicle for reaching young people in SSA in their communities. Concurrently, enhancing MHL capacity in schools through curriculum embedded resources integrated educational activities) can enhance MHL for both teachers and students alike (35, 36–38).

Enhancing MHL is a component of the Integrated Approach to Addressing the Issue of Youth Depression (IACD) in Malawi and Tanzania. This was achieved in the community by youth-participant interactive radio broadcasts and in schools through teacher training and application of an MHL curriculum resource and school-based radio-listening clubs. Peer mental health educator training was also used in Malawi.

## Youth-Engaging Radio Programs

Stigma and lack of awareness about mental disorders are cited as a significant barrier to help seeking in SSA (14, 20, 39). In order to address these issues on a mass scale in communities, we designed and implemented interactive, weekly radio programs that combined radio dramas, quizzes, polls, and “ask the expert” phone-ins.

The design of these was based on formative research with young people, which assessed their current level of knowledge, awareness, and attitudes regarding mental health/mental disorders, as well their radio-listening habits and preferences. While a detailed description of the methods applied and results obtained is beyond the scope of this descriptive report and will be separately published, over 4,000 youth in both Malawi and Tanzania participated in a community survey that provided baseline data that was used to inform the design and content of the programs. Additionally, input was sought from local mental health experts and youth to create content that was accurate, appropriate, and attractive for the target audience. Called “Nkhawa Njee” in Malawi and “Positive Mood” in Tanzania, the programs aired on radio stations popular with young people. Other than a serialized, short (approx. 4–5 min per episode) soap opera, the weekly program was a freestyle show hosted live every week and, in addition to community broadcast, was linked to schools in which mental health literacy training and curriculum resources had been applied (see below) through weekly radio-listening clubs. Teachers trained in the MHL curriculum and trained peer mental health educators facilitated discussions about issues raised by the radio program.

The radio program was evaluated using a randomized cluster design that compared changes in young people’s scores for knowledge, attitudes, and mental health seeking efficacy in radio “target” areas with control areas. Results show significant improvements in young people who listened to the program than in those who had no exposure to it (http://mhinnovation.net/innovations/integrated-approach-addressing-issue-youth-depression#.V5ZZ_7fdXcs).

## School-Based Approaches

School-based approaches consisted of four components: 1) training teachers on the use of an MHL curriculum resource (40) that they could then use to teach students in usual classroom settings; 2) establishing school-based listening clubs (in both Malawi and Tanzania) enriched by training of peer mental health educators (Malawi only); 3) training teachers on how to identify youth showing signs and symptoms of depression; and 4) facilitating referral from schools to local community health-care providers.

The teacher training was based on the adaptation of an evidence-based Canadian school mental health literacy resource, the Guide, freely available at www.teenmentalhealth.org (34, 36–38), adapted by mental health and education experts selected by the Ministries of Health in Malawi and Tanzania to create the African Guide, which was then taught to teachers by master trainers expert in mental health and education in both countries. Thirty-five schools (a mixture of public, private, religious, boarding, and day schools) identified by the Ministries of Education and District Educational authorities were chosen as test sites in each country. Master trainers provided training to two to three teachers per school as selected by school headmasters. Research studies evaluating improvements in teacher’s mental health literacy using prospective cohort designs were conducted in both countries. Teacher refresher training was conducted about 6 months following initial training. This intervention demonstrated positive impacts with significant, substantial, and sustained improvements in all aspects of teacher’s mental health literacy, including improved knowledge, decreased stigma, and enhanced help-seeking personally and for friends and family members (40) as well as the application of this resource into classroom settings with positive outcomes (41–43).

### Community Health-Care Provider Competence Development in Identification, Diagnosis, and Treatment of Youth With Depression

Most community-based health-care providers have received little or no training in youth mental health care in either Malawi or Tanzania. A Canadian youth Depression training program (certified by the Canadian College of Family Practice, one of the courses offered by MDcme.ca) was adapted for use in Malawi and Tanzania by mental health experts including psychiatrists, psychologists, and psychiatric nurses. All materials were translated into Chichewa (Malawi) and Kiswahili (Tanzania). Community health-care clinics were chosen by the Ministries of Health and District Health authorities for participation in the project. Master trainers in both countries were trained by the principle investigator (SK) in the use of the training program. Master trainers then trained a cadre of health-care providers from secondary-level settings who then trained health-care providers working in community health clinics in both an initial training session and a refresher course 6 months later. Prospective longitudinal cohort studies of provider knowledge, stigma, and self-confidence were conducted. Results obtained demonstrated significant positive impact of the intervention in all these domains (43–46).

In addition, patient data collection forms were created and provided to clinicians in community health centers who had received the training program. Data collected included screening for depression, numbers diagnosed, type of treatment provided, and patient outcomes. Outcomes showed both feasible and positive impacts of the intervention on care delivery and patient outcomes (44).

All clinical care providers were also trained on a psychotherapeutic intervention comprising counseling and cognitive behavioral therapy techniques developed for use by the principle investigator (SK) and a colleague expert psychotherapist (Dr. Susana Costa, Lisbon, Portugal) and designed to be used by community health-care providers to enhance non-pharmacologic treatment competencies. Trainer and provider respondent impressions of the utility and applicability of this intervention are being collected with the goal of further modification and later evaluation of the impact of this type of psychological intervention on patient outcomes in these settings.

## Discussion

To our knowledge, this is the first reported application of a horizontally integrated pathway to youth mental health care for depression in any low- or medium-income country. Results of all components analyzed to date show significant positive impacts in both the initial Malawi application and the Tanzania replication, suggesting that this innovative merging of mental health literacy development at both community (through radio programs) and schools (through teacher training and radio-listening clubs), case identification, and linking of schools to community clinics (supported by competency-based training in diagnosis, and treatment of youth depression at community clinics) is ready for mental health policy application in both counties and could be considered for scale out in SSA.

While this innovation may now be considered for policy-directed implementation, further study of the implementation process may also be indicated to provide a better understanding of the factors that affect policy development and innovation implementation. By comparing and contrasting the policy development and implementation of this innovation in these two different settings, it may be possible to better appreciate which aspects are generalizable and relatively easily transferred from one context to another and which may require adaptation.

We are aware that this policy-informing approach has a number of limitations. One is the ability of the Ministry of Health in any low-income country to allocate necessary resources towards youth mental health care. While funding for this work has been provided by Grand Challenges Canada, this revenue source is finite. At some point, the government will need to take on the funding challenge as well as developing the policy framework that it will sustain. Perhaps the recent announcement of the World Bank as a third-party funding source for assisting LMICs in addressing mental health-care needs will be a help with this issue (http://www.who.int/mental_health/WB_WHO_meeting_2016.pdf). As well, in both countries, the Ministries of Health have modified existing plans and resource allocations to better provide interventions and some of the resources needed to address the therapeutic needs of youth with depression.

A further set of challenges will be related to the need for Ministries of Health and Education to collaborate in the application of this innovation—at both policy and implementation levels. How to best provide MHL training to teachers in schools and how to best link schools to local community health centers will require input from both.

These cross-ministry collaborations may also need to include consideration of embedding some of the training programs into institutions that currently train health-care providers and teachers, so that the necessary care competencies can be taught in pre-service curriculum of providers and MHL to pre-service teachers. It is encouraging that to date, in both countries, the Ministries of Health (with additional funds from Grand Challenges Canada) decided to collaborate with the implementation team to field test this approach in one nursing college per country, and the results of this intervention from Malawi are currently being analyzed.

Due to difficulties in maintaining patient records at many community health centers in LMICs, it may be difficult at this time to draw firm conclusions about changes in individuals’ health outcomes. We used a real world rather than a regulated test site approach to addressing this issue; thus, our results are not as pristine as those obtained from data collected in a controlled setting. On the other hand, these data are a better reflection of what is possible on the ground than is the relative serenity of a designated research setting. We anticipate difficulties with health provider compliance in updating patient tracking forms on a regular basis, as well as relatively high levels of attrition of young people receiving treatment due to lack of transportation, long wait times at clinics, and other factors that may compromise patient care as well as data collection. We do anticipate, however, that our current work will highlight barriers and provide suggestions for improving patient monitoring activities.

## Conclusion

Taken as a whole, this evidence-based innovation applied in Malawi and replicated in Tanzania can be used to potentially inform the development of mental health policy and interventions across SSA. Such impact has already begun in both of these countries. However, we realize that there may be numerous factors at play that may prevent or limit this application. Future research will be necessary to identify both those factors that facilitate and those that impede such policy development.

## Data Availability

Data will be provided upon request.

## Ethics Statement

Ethics approval for all the Tanzania studies was received from the National Institute for Medical Research and the Ministry of Health. In Malawi, ethics approval was obtained through the Guidance, Counselling and Youth Development Centre for Africa from the Ministry of Education.

In the field interview studies of radio-listening feedback and training evaluations, verbal consent to participate was obtained from respondents, and no individual-identifying data were obtained. In the evaluation of health system studies, no individual written consent was required, as the studies used data from clinic reporting that had no individual identification.

## Author Contributions

SK was the principle investigator and creator of most of the educational resources used in this project. He also provided oversight of all data entry, analysis, and interpretation. KP is the director of Farm Radio International and co-investigator in the project. He also directed financial oversight and audit. HG was the project coordinator and was responsible for the on-the-ground application of all aspects of the project in both countries. MU was the lead on the Malawi component of the project. He also directed adaptation of all materials and training programs for Malawi. OU was the co-lead on the Tanzania component of the project. He also directed adaptation of all materials and training programs for Tanzania. TN was the co-lead on the Tanzania component of the project. She also directed adaptation of all materials and training programs for Tanzania. RC was the director of Farm Radio Malawi. He directed the Malawi application of the radio intervention. MH is a research assistant who was responsible for manuscript oversight, preparation, and submission.

## Funding

Funding to support the project from which this study was generated was provided by Grand Challenges Canada (Grant Number 0090-04).

## Conflict of Interest Statement

The authors declare that the research was conducted in the absence of any commercial or financial relationships that could be construed as a potential conflict of interest.

## References

[1] World Health Organization The world health report 2001. In: Mental health: new understanding, new hope. Geneva: World Health Organization (2001).

[2] World Health Organization The global burden of disease: 2004 update. Geneva: World Health Organization (2008). Retrieved from http://www.who.int/healthinfo/global_burden_disease/GBD_report_2004update_full.pdf?ua=1.

[3] Kutcher S Venn D Why youth mental health is so important. Medscape J Med (2008) 10:275. 19242581 PMC2644010

[4] Kessler RC Berglund P Demler O Jin R Merikangas KR Walters EE Lifetime prevalence and age-of-onset distributions of DSM-IV disorders in the National Comorbidity Survey Replication. Arch Gen Psychiatry (2005) 62:859–77. 10.1001/archpsyc.62.6.593 15939837

[5] Prince M Patel V Saxena S Maj M Maselko J Phillips MR No health without mental health. Lancet. (2007) 370:859–77. 10.1016/S0140-6736(07)61238-0 17804063

[6] Patel V Flisher AJ Hetrick S Mcgorry P Mental health of young people: a global public-health challenge. Lancet. (2007) 369:1302–13. 10.1016/S0140-6736(07)60368-7 17434406

[7] Patel V Saxena S Transforming lives, enhancing communities—innovations in global mental health. N Engl J Med (2014) 370:498–501. 10.1056/NEJMp1315214 24428425

[8] McEwan K Waddell C Barker J Bringing children’s mental health ‘out of the shadows’. CMAJ (2007) 176(4):471–72. 10.1503/cmaj.061028 PMC1800563 17296959

[9] Chisholm D Sweeny K Sheehan P Rasmussen B Smit F Cuijpers P Saxena S Scaling-up treatment of depression and anxiety: a global return on investment analysis. Lancet Psychiatry (2016) 3:415–24. 10.1016/S2215-0366(16)30024-4 27083119

[10] Nduna M Jewkes R Dunkle K Jama Shai N Colman I Prevalence and factors associated with depressive symptoms among young women and men in the Eastern Cape Province, South Africa. J Child Adolesc Men Health (2013) 25:43–54. 10.2989/17280583.2012.731410 25860306

[11] Cortina M Sodha A Fazel M Ramchandani P Prevalence of child mental health problems in sub-Saharan Africa: a systematic review. Arch Pediatr Adolesc Med (2012) 166:276–81. 10.1001/archpediatrics.2011.592 22393184

[12] Belfer ML Child and adolescent mental disorders: the magnitude of the problem across the globe. J Child Psychol Psychiatry (2008) 49(3):226–36. 10.1111/j.1469-7610.2007.01855.x 18221350

[13] Udedi M The prevalence of depression among patients and its detection by primary health care workers at Matawale Health Centre (Zomba). Malawi Med J (2014) 26:34–7. PMC4141239 25157314

[14] Kauye F Chiwamdira C Wright J Increasing the capacity of health surveillance assistants in community mental health care in a developing country. Malawi Med J (2011) 23:85–8. PMC3588564 23448002

[15] Stewart R Umar C Tomenson E Creed B A cross-sectional study of antenatal depression and associated factors in Malawi. Arch Womens Ment Health (2014) 17:145–54. 10.1007/s00737-013-0387-2 24240635

[16] Kim M Mazenga A Devandra A Ahmed S Kazembe P Yu X Prevalence of depression and validation of the Beck Depression Inventory-II and the Children’s Depression Inventory-Short amongst HIV-positive adolescents in Malawi. J Int AIDS Soc (2014) 17:18965–77. 10.7448/IAS.17.1.18965 PMC4119288 25085002

[17] Nyandini US Tanzania global school-based student health survey report. Retrieved from World Health Organization (2008). Online: http://www.who.int/chp/gshs/TANZANIA_GSHS_FINAL_REPORT_2008.pdf

[18] Fatiregun AA Kumapayi TE Prevalence and correlates of depressive symptoms among in-school adolescents in a rural district in southwest Nigeria. J Adolesc (2014) 37:197–203. 10.1016/j.adolescence.2013.12.003 24439625

[19] Khasakhala LI Ndetei DM Mathai M Harder V Major depressive disorder in a Kenyan youth sample: relationship with parenting behavior and parental psychiatric disorders. Ann Gen Psychiatry (2003) 12(5):1–10. 10.1186/1744-859X-12-15 PMC3660220 23663452

[20] Crabb J Stewart RC Kokota D Masson N Chabunya S Krishnadas R Attitudes toward mental illness in Malawi: a cross-sectional survey. BMC Pub Health (2012) 12:541. 10.1186/1471-2458-12-541 22823941 PMC3413535

[21] Hugo C Boshoff J Traut D Zungu-Dirwayi E Stein L Community attitudes toward and knowledge of mental illness in South Africa. Soc Psychiatry Psychiatr Epidemiol (2003) 38:715–19. 10.1007/s00127-003-0695-3 14689176

[22] World Health Organization Mental health atlas. In: United Republic of Tanzania. Geneva World Health Organization (2014).

[23] Muula A Kazembe L Rudatsikira E Siziya S Suicidal ideation and associated factors among in-school adolescents in Zambia. Tanzan Health Res Bull (2007) 9:202–6. 10.4314/thrb.v9i3.14331 18087900

[24] World Bank Data: Malawi. Washington: World Bank (2014).

[25] World Health Organization (2011). Mental health atlas. Retrieved from United Republic of Tanzania: http://www.who.int/mental_health/evidence/atlas/profiles/tza_mh_profile.pdf?ua=1.

[26] World Bank Poverty headcount ratio at $1.25 a day (PPP). Washington: World Bank (2014).

[27] World Health Organization The World Health report 2006: working together for health. Geneva: World Health Organization (2006). (http://www.who.int/whr/2006/en.

[28] Bartlett P Jenkins R Kiima D Mental health law in the community: thinking about Africa. Int J Ment Health Syst (2011) 5(1):1. 10.1186/1752-4458-5-21 21914182 PMC3189124

[29] Jorm AF Korten AE Jacomb PA Christensen H Rodgers B Pollitt P “Mental health literacy”: a survey of the public’s ability to recognise mental disorders and their beliefs about the effectiveness of treatment. Med J Aust (1997) 166:182–6. 10.5694/j.1326-5377.1997.tb140071.x 9066546

[30] Reavley NJ Jorm AF National survey of mental health literacy and stigma. Canberra: Department of Health and Ageing (2011). (http://pmhg.unimelb.edu.au/research_settings/general_community/?a=636496). 10.3109/00048674.2011.621061 22023236

[31] Jorm AF Mental health literacy: empowering the community to take action for better mental health. Am Psychol (2012) 67:231–43. 10.1037/a0025957 22040221

[32] Wei Y Hayden J Kutcher S Zygmunt A McGrath P The effectiveness of school mental health literacy programs to address knowledge, attitudes and help seeking among youth. Early Interv Psychiatry (2013) 7:109–21. 10.1111/eip.12010 23343220

[33] Kutcher S Wei Y School mental health literacy: a national curriculum guide shows promising results. Educ Can (2014) 54:22–6.

[34] Kutcher S Bagnell A Wei Y Mental health literacy in secondary schools: a Canadian approach. Child Adolesc Psychiatr Clin N AM (2015) 24:233–44. 10.1016/j.chc.2014.11.007 25773321

[35] Kutcher S Wei Y Coniglio C Mental health literacy: past, present, and future. Can J Psych (2016) 61:154–58. 10.1177/0706743715616609 PMC4813415 27254090

[36] McLuckie A Kutcher S Wei Y Weaver C Sustained improvements in students’ mental health literacy with use of a mental health curriculum in Canadian schools. BMC Psychiatry (2014) 14:379. 10.1186/s12888-014-0379-4 25551789 PMC4300054

[37] Milin R Kutcher S Lewis SP Walker S Ferrill N Randomized controlled trial of a school-based mental health literacy intervention for youth: impact on knowledge, attitudes and help-seeking efficacy. 60th Annual Meeting AACAP (2013).

[38] Milin R Kutcher S Lewis SP Walker S Wei Y Ferrill N Impact of a mental health curriculum for high school students on knowledge and stigma: a randomized controlled trial. J Am Acad Child Adolesc Psychiatry (2016) 55:383–91. 10.1016/j.jaac.2016.02.018 27126852

[39] Mbatia J Shah A Jenkins R Knowledge, attitudes and practice pertaining to depression among primary health care workers in Tanzania. Int J Ment Health Syst (2009) 3:5. 10.1186/1752-4458-3-5 19243596 PMC2652424

[40] Kutcher S Gilberds H Morgan C Greene R Hamwaka K Perkins K Improving Malawian teachers’ mental health knowledge and attitudes: an integrated school mental health literacy approach. Global Ment Health (2015) 2:1–10. 10.1017/gmh.2014.8 PMC4964842 28596850

[41] Kutcher S Wei Y Gilberds H Brown A Ubuguyu M Njau T The African guide: one year impact and outcomes from the implementation of a school mental health literacy curriculum resource in Tanzania. J Educ Studies (2017) 5(4):64–73. 10.11114/jets.v5i4.2049

[42] Kutcher S Wei Y Gilberds H Brown A Ubuguyu O Njau T Evaluating community health care providers knowledge and self-confidence in the identification, diagnosis and treatment of adolescent depression in Tanzania. Arch Depress Anxiety (2016) 2(1):23–30. 10.17352/2455-5460.000011

[43] Kutcher S Gilberds H Morgan C Udedi M Perkins K Malawi educators’ assessment of student mental health outcomes. Int J Sch Cogn Psychol (2015) SC2009. 10.4172/2469-9837.1000S2-009

[44] Kutcher S Wei Y Gilberds H Ubuguyu O Njau T Brown A A school mental health literacy curriculum resource training approach: effects on Tanzanian teachers’ mental health knowledge, stigma and help-seeking efficacy. Int J Ment Health Syst (2016) 10:50. 10.1186/s13033-016-0082-6 27493684 PMC4973111

[45] Kutcher S Wei Y Gilberds H Brown A Ubuguyu O Njau T Addressing adolescent depression in Tanzania: Positive primary care workforce outcomes using a training cascade model. Depress Res Treat (2017) 2017(2017):9. 10.1155/2017/9109086 PMC5733241 29333294

[46] Kutcher S Udedi M Gilberds H Brown A Chapota R Perkins K Clinic outcomes of the pathway to care model: a cross-sectional survey of adolescent depression in Malawi. Malawi Med J (2017) 29(2):97–102. 10.4314/mmj.v29i2.4 28955414 PMC5610277

